# Discovery of RNA Biomarkers for Prostate Cancer Using Cross-Platform Transcriptomics

**DOI:** 10.3390/ijms252211907

**Published:** 2024-11-06

**Authors:** Wieke C. H. Visser, Hans de Jong, Frank P. Smit, Jolly Shrivastava, Jason C. Poole, William P. J. Leenders, Willem J. G. Melchers, Peter F. A. Mulders, Jack A. Schalken

**Affiliations:** 1 Department of Product Development, Mdxhealth BV, 6534 AT Nijmegen, The Netherlands; 2 Department of Product Development, Mdxhealth, Irvine, CA 92618, USA; 3 Predica Diagnostics, 6525 ED Nijmegen, The Netherlands; 4 Department of Medical Microbiology, Radboud University Medical Center, 6525 GA Nijmegen, The Netherlands; 5 Department of Urology, Radboud University Medical Centre, 6525 GA Nijmegen, The Netherlands

**Keywords:** microarray, single-molecule molecular inversion probes (smMIPs), prostate cancer (PCa), gene expression profiling, biomarkers

## Abstract

Microarray and Single-Molecule Molecular Inversion Probe (smMIP)-based targeted RNA sequencing are two RNA profiling platforms for identifying disease-associated biomarkers. The microarray uses a GeneChip array with oligonucleotide probes to measure expression levels across thousands of genes, while smMIPs capture and quantify RNA transcripts and transcript variants via next-generation sequencing. To evaluate the strengths and weaknesses of both platforms, a comparative gene expression profiling study was conducted using RNA samples from 52 prostate tissues (normal, benign prostatic hyperplasia (BPH) and various prostate cancer (PCa) grades). Of all genes covered by both platforms, only 35% of the expression levels aligned, with 45% showing discrepancies. Both platforms identified the same 17 genes as potential PCa biomarkers. Microarray analysis identified an additional 253 genes that were not covered or not identified by smMIP technology, while smMIP technology identified eight markers not covered or not identified in the microarray core gene analysis, including fusion genes and splice variants. For high-grade prostate cancer (HG-PCa), the smMIP-method identified 8 markers, and the microarray identified 17 markers, with *FOLH1, FAP* and *CLDN3* being common across both platforms. The choice of RNA expression analysis technology depends on research objectives; microarray technology is useful for the evaluation of a wide range of genes but has low throughput. In contrast, smMIP-based RNA sequencing enables sensitive analysis with minimal RNA in a medium- to high-throughput setting.

## 1. Introduction

Over the last decades, various platforms have been developed to identify and validate potential disease-associated biomarkers. In the present study, the microarray platform was compared to Single-Molecule Molecular Inversion Probe (smMIP)-based targeted RNA sequencing technology to analyze gene expression profiles associated with prostate cancer (PCa). The microarray platform is a widely used platform for RNA profiling [1]. The platform incorporates a GeneChip array containing oligonucleotide probes with sequences complementary to sequences of genes of interest, allowing for the generation of gene expression profiles of thousands of genes [2].

A more recent and innovative approach for RNA profiling is smMIP-based targeted RNA sequencing [3,4]. The approach uses molecular inversion probes designed to capture and copy specific regions of cDNAs of interest that can be quantitatively measured using next-generation sequencing. The targeted character of the technology requires relatively little sequencing capacity per sample, allowing parallel processing of many hundreds of different samples using barcoding technology and resulting in low sequencing costs per sample. The number of genes of interest that can be measured in individual samples can range up to thousands. Next to that smMIP-based sequencing allows for the detection of clinically relevant transcript variants and gene mutations [3,4]. An obvious disadvantage of targeted approaches like microarray and targeted smMIP sequencing is that they require prior knowledge. Although the targeted approach of both methods affects the possibility of discovering new biomarkers, both methods still offer the possibility of identifying biomarkers that are already known but have not yet been associated with specific diseases.

Following the recent tendency towards sequencing-based technologies for RNA expression analysis, there is a need for a direct comparison with traditional microarray technology. In this study, we performed a head-to-head comparison between smMIP-based targeted RNA sequencing and Affymetrics microarray gene expression analysis, using a cohort of prostate samples with different histopathology diagnoses.

## 2. Results

### 2.1. Correlation of Gene Expression Levels Across Platforms

**Figure 1:**
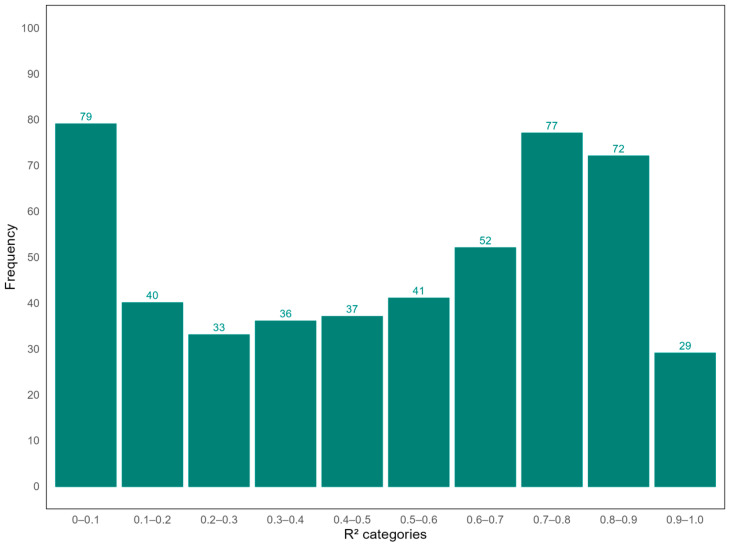
Distribution of correlation values (R^2^) of the correlation of RNA expression levels between smMIP and the microarray. The figure presents the frequency distribution of R^2^-values reflecting the correlation between gene expression measurements across the set of genes that were measured with both the smMIP platform and the microarray platform. In total, expression levels of 496 genes were measured on both platforms. Good correlations were observed for 178 genes (35.89%), with R^2^ > 0.7. Moderate correlations (R^2^ between 0.5 and 0.7) were observed for 93 genes (18.75%) and weak correlations (R^2^ < 0.5) were shown for 225 genes (45.36%).

Overall, the microarray analysis evaluated over 22,000 genes, whereas the smMIP-based method covered 688 genes. In total, 496 genes were covered by both the microarray method and the smMIP-based RNA sequencing platform. To assess the concordance in gene expression levels measured by both platforms, R^2^-values were calculated. Overall, the correlation between the two methods varied considerably across genes. Good correlations were observed for 178 genes (35.89%, with R^2^-values > 0.7). Moderate correlations (R^2^-values between 0.5 and 0.7) were observed for 93 genes (18.75%) and weak correlations (R^2^-values < 0.5) were seen for 225 genes (45.36%) (Figure 1).

Further analysis was carried out to explore the reasons behind the weak correlation observed for 225 genes. For 170 of these genes, the weak correlation could be attributed to expression levels being below the detection limits of at least one platform, defined as 2log values ≤ 6 for the microarray or 2log values ≤ 1.0 for the smMIP-based method. Another factor contributing to the poor correlations is the low variation in expression levels between samples for certain genes as detected with either one of the techniques.

### 2.2. Biomarker Identification

To compare the potential biomarkers that were identified across the two techniques, two analyses were performed. First, gene expression levels in PCa tissue were compared to normal prostate (NP) and BPH samples. Secondly, gene expression levels in HG-PCa tissue were compared to normal prostate, BPH and low-grade prostate cancer (LG-PCa) samples.

#### 2.2.1. Biomarkers to Detect PCa

The smMIP analyses identified 27 genes exhibiting at least a twofold higher 2log value in PCa tissue compared to no PCa tissue. Among these, 25 genes demonstrated significantly elevated expressions in PCa in comparison to no PCa (*p*-value < 0.05, Table 1). Microarray analysis detected 271 genes with at least a fold change exceeding 2, of which 270 showed a significant *p*-value (Table 2 and Supplementary Table S1).

Overall, 17 out of the 25 genes identified as significant by the smMIP analysis were also identified by microarray analysis. These genes include *EPCAM, DLX1, ODC1, PLA1A, SOX14, EGF, CLDN4, CLDN3, MYC, FBP1, FOLH1, HOXC6, SLC7A1, ALOX15, TOP2A, FASN* and *ERG*. The smMIP analysis identified potential biomarkers that were not covered in the microarray core gene analysis (*LNCHOXC8, PCA3* and *TMPRSS2-ERG*) or were not identified as potential markers by microarray analysis (*BIRC5, CD24, EZH2, GLDC* and *IL6*). On the other hand, the microarray analysis identified 250 genes as markers not included in smMIPs (see Supplementary File S2), or not recognized as potential markers by the smMIP analysis (*LYPLA1, RUVBL2* and *SLC5A1*).

#### 2.2.2. Biomarkers to Detect HG-PCa

The comparison of the two RNA expression analysis methods extended to the identification of HG-PCa, comparing HG-PCa to either no PCa or LG-PCa. The smMIP analysis identified 14 genes with a 2log value difference greater than 2 between HG-PCa versus no PCa and LG-PCa, of which 8 genes showed significantly higher expression levels in HG-PCa as compared to the no PCa/LG-PCa group (adjusted *p*-value < 0.05, Table 3). The microarray analysis showed 17 targets with a significantly higher expression level in HG-PCa and a fold change greater than 2 (adjusted *p*-value < 0.05, Table 4).

The genes *FAP, CLDN3* and *FOLH1* are promising markers for HG-PCa identified with both platforms. One marker was significant in the smMIP analysis, but not found in the microarray as this gene was not covered in the microarray (*LNCHOXC8*). Additionally, markers *ALOX15, BIRC5, CLDN4* and *HOXC6* were covered in both methods, but only significant in the smMIPs analysis for HG-PCa. Focusing on the microarray, 14 potential markers identified by the microarray were not covered by the smMIPs (*CHRM3, RRM2, AMACR, TRIB1, ASPN, RPL35, MS4A8, PPFIA2, MYL2, ITGBL1, SHISA2, COMP, CLDN8* and *ND6*).

## 3. Discussion

This comparative report presents the results of biomarker discovery for PCa by comparing gene expression analysis between the microarray and smMIP-based platform. R^2^ values were calculated to evaluate the correlation between expression levels measured by both methods. Subsequent to that, potential biomarkers for PCa and HG-PCa identified using both techniques were analyzed, including comparisons between PCa and no PCa, as well as between HG-PCa and LG-PCa or no PCa.

The first approach is to compare both techniques involved in the evaluation of the correlation between RNA expression levels measured by each method. A total of 496 genes were covered with both techniques. Expression levels of a subset of these genes (~35%) were similar when comparing these levels between both techniques. However, for a major part of genes (~45%), RNA expression levels did not align between the two techniques. This weak correlation resulted in differences in identifying genes as potential biomarkers, as some genes were considered significant biomarkers using only one of the methods. The poor correlation in expression levels of this subset of genes between both methods could be attributed to several factors: variations in assay efficiency, differences in probe(s) location within the target of interest, differences in the number of probes used per target (smMIP technology includes up to five probes per target, whereases the microarray can include dozens of probes per target) and the difference in sensitivity for detecting the RNA transcripts of interest of both methods. However, this discrepancy did not show a clear preference for one of the methods, as the variation in performance was observed in both techniques. In an earlier study, Zhao and co-workers [5] conducted a comparison between microarray and total RNA sequencing approaches in T cell research. The research showed the superiority of RNA sequencing over microarray in detecting isoforms and identifying variants. Furthermore, they demonstrated a broader dynamic range in RNA sequencing and a strong correlation, the latter being observed in only a subset of the genes assessed in our analysis.

Subsequent to the correlation analysis, both techniques were compared to biomarker discovery results. Overall, the microarray covered a significantly larger number of targets compared to the smMIP-based platform, resulting in the identification of many more potential biomarkers with microarray. Most potential biomarkers identified with smMIP-based analysis were also identified with the microarray. Although these results suggest no clear added value of using smMIPs over microarray analysis, there are advantages to using smMIPs in biomarker research. The smMIP-based technology enables transcript analysis for biomarker discovery in samples with low amounts of RNA (the minimal input of RNA for smMIPs is 30 ng compared to 1 µg for the microarray) and it has the capacity to analyze gene mutations and variants such as alternative gene splicing (known to be associated with cancer [6]) and fusion transcripts. This is also observed in the current analysis, where the *TMPRSS2-ERG* fusion transcript (widely known to be associated with PCa [7]) was confirmed by smMIP analysis as a potential PCa biomarker, while this fusion transcript was not covered by the microarray. Next to that, although most markers identified by smMIP analysis can also be found with a microarray, the smMIP technique still identified novel potential markers, for example, *BIRC5, CD24, EZH2, GLDC* and *IL6*. These genes were all previously described to play a role in PCa [8,9,10,11,12].

The microarray analysis described in this paper is based on the core probe set, containing the most reliable and annotated probes. However, the microarray chip also includes probes with less experimental evidence, including poorly annotated sequences that were still under study at the time or probes developed using prediction algorithms. The microarray has evolved over the past years, resulting in a new version of the gene chip (Affymetrix™ GeneChip™ Human Transcriptome Array 2.0, Thermo Fisher Scientific, Santa Clara, CA, USA). This gene chip includes over six million probes, also covering long non-coding RNA transcripts. Therefore, a limitation of this study is that only the results from the core probe set of the initial 1.0 gene chip were evaluated, which could explain some of the differences in potential biomarkers found with the smMIP-based platform as compared to the microarray. For example, *PCA3* is one of those genes, which was not included in the core probe set but which was included in the microarray full probe set as well as in the smMIP panel. The smMIP analysis confirmed *PCA3* as a biomarker. In total, there were 191 genes that were covered with the smMIP panel which were not covered in this microarray analysis (core probe set). Next to *PCA3*, *TMPRSS2-ERG* (described earlier) and *LNCHOXC8* were identified only by the smMIP-based platform as potential markers. *LNCHOXC8* is a VISTA enhancer hs2078 which is a regulatory element that plays a role in controlling gene expression. VISTA enhancer hs2078 is a 3208 bp long DNA sequence that is in the genomic region of the HOXC cluster. Aberrant expressions of genes from the HOXC cluster are associated with PCa [7]. Enhancers are often part of the non-coding genome, and therefore, this target is not included in the core probe set analysis of the microarray.

## 4. Materials and Methods

### 4.1. Sample Collection and RNA Purification

Sample collection and RNA purification procedures were previously performed [13,14]. NP and LG-PCa tissue samples were obtained during radical prostatectomy (RP). BPH tissue samples were obtained at transurethral resection of the prostate (TURP). HG-PCa tissue samples were obtained either during RP or TURP, as not all patients with HG-PCa underwent RP. Following this, samples were snap-frozen, sectioned and stained with hematoxylin and eosin for histological classification. After dissection of the tumor and tumor-free areas, total RNA was extracted using TRIzol Reagent (Invitrogen, Carlsbad, CA, USA). Following, samples were DNase-treated and purified using the RNeasy kit from Qiagen (Qiagen, Hilden, North Rhine-Westphalia, Germany, 40724). RNA quantity and quality were examined using a Nanodrop Instrument and an Agilent 2100 Bioanalyzer. Samples with an RNA integrity number (RIN) ≥ 6 were included for further analysis [6]. For this study, prostate tissue samples of NP (*n* = 7), BPH (*n* = 11), LG-PCa (*n* = 17, defined as Gleason Score < 7) and HG-PCa (*n* = 17, defined as Gleason Score ≥ 7) were selected for analysis based on the availability of RNA samples.

### 4.2. Microarray

Microarray RNA profiling data were obtained using GeneChip Human Exon 1.0 Sense Targets microarrays (Affymetrix, Santa Clara, CA, USA) as described before [13,14]. In total, 1 µg of total RNA was used in the Affymetrix GeneChip Whole Transcript Sense Target Labeling Assay (Affymetrix, Santa Clara, CA, USA) to generate cDNA. Through in vitro transcription, a large amount of cRNA molecules were generated. Following this, cDNA synthesis was performed followed by fragmentation and biotinylation. The final amplified, fragmented and biotinylated sense-strand cDNA was inserted into a hybridization mixture, which was transferred to the GeneChip Human Exon 1.0 Sense Targets microarray (Affymetrix, Santa Clara, CA, USA). The GeneChip included over 5 million oligonucleotide probes, with over 20 probes per gene [13]. In total, over 22,000 genes were covered. After hybridization, a washing and staining step was performed. Finally, the GeneChips were scanned using a GeneChip Scanner (Affymetrix, Santa Clara, CA, USA). The Partek Genomic Suite (Version 6.6, Partek Incorporated, St. Louis, MI, USA) was used to normalize the data across arrays, to perform background correction and to summarize the data. Final gene expression levels were reported as 2log values [14].

### 4.3. Targeted Sequencing with smMIPs

cDNA was generated from the same purified, total RNA that was used for microarray analysis. SmMIP-based RNA expression profiles were obtained by targeted sequencing using smMIPs as described earlier [4]. The smMIP panel was designed to detect gene transcripts associated with cancer cell biology, including transcription factors, proto-oncogenes, tumor suppressor genes and genes involved in metabolism, cell migration and cell cycle processes. Subsequent to that, the panel was supplemented with smMIPs targeting prostate-specific targets (e.g., prostate-specific fusion gene *TMPRSS2-ERG*). cDNAs were captured with a final pool of 2808 smMIPs for the detection of 688 gene transcripts according to previously published protocols [3,4]. Barcoded libraries were sequenced on an Illumina Novaseq6000 SP flow cell. The average sequencing depth per sample, expressed as total reads, was 853,706 (median 871,458 (63,101–1,330,954)). In-house built software was used to process FASTQ files to normalized gene expression levels (expressed as Fragment Per Million (FPM) values), followed by a 2log transformation.

### 4.4. Statistical Analysis

Statistical analyses were performed using RStudio (Version 4.4.0, RStudio, PBC, Boston, MA, USA). All statistical analyses were performed on both 2log-transformed FPM values (smMIP-based platform) and on 2log expression levels from the microarray. Linear regression analysis was performed to evaluate the correlation between expression levels measured by both methods, resulting in R^2^ values to quantify the variance. For the identification of potential PCa biomarkers, the 2log values of several groups were compared using the Mann–Whitney U test. First, mean 2log values were compared between NP + BPH and LG-PCa + HG-PCa. Secondly, mean 2log values were compared between NP + BPH + LG-PCa and HG-PCa. The Benjamini–Hochberg method was applied to adjust *p*-values for multiple testing sessions. A significance level of 0.05 was used for all statistical tests. The results of the Mann–Whitney U test were used to compare the biomarkers identified by the microarray with those identified by the smMIP method.

## 5. Conclusions

Selecting the most appropriate technology for RNA expression analysis depends on the specific research objectives. Overall, microarray technology is ideal for broad biomarker discovery across approximately 22,000 genes, while smMIPs are better suited for focused analyses on limited RNA samples or specific genetic modifications, such as cancer-associated fusions or variants. In more detail, microarray technology provides advantages for studies aiming to evaluate expression levels of a wide range of genes, as this technology enables the analysis of transcripts of approximately 22,000 genes. However, its low-throughput capacity can limit the utility of the microarray. On the other hand, when studying a selected set of transcripts in a medium- to high-throughput manner, smMIP-based RNA sequencing enables sensitive RNA transcription analysis requiring a relatively small amount of RNA. Overall, both methods require prior knowledge. For the identification of new biomarkers without the availability of prior knowledge, a non-targeted approach such as total RNA sequencing is required.

## Figures and Tables

**Table 1:** The results of smMIP analysis for the 25 genes with expression levels that are significantly higher in PCa compared to no PCa and with a fold change > 2.

	Mean FPM Log2 per Group			
Gene ^1^	No PCa (Normal and BPH)	PCa (LG-PCa and HG-PCa)	Fold Change ^2^	Adjusted *p*-Value ^3^
*PCA3*	3.13	9.60	88.78	0.00
*TMPRSS2-ERG*	0.00	4.61	24.37	0.00
***HOXC6***	1.83	5.14	9.91	0.00
***CLDN3***	2.47	5.32	7.20	0.00
***DLX1***	0.72	3.53	7.01	0.00
***ERG***	5.70	8.14	5.42	0.03
*LNCHOXC8*	0.90	3.30	5.26	0.00
***PLA1A***	5.53	7.71	4.53	0.00
***ALOX15***	0.20	2.32	4.34	0.00
***FASN***	6.75	8.85	4.30	0.00
***FOLH1***	10.48	12.56	4.23	0.00
***MYC***	7.89	9.91	4.04	0.00
***SOX14***	2.54	4.24	3.24	0.02
***CLDN4***	5.76	7.44	3.20	0.00
*IL6*	4.66	6.30	3.13	0.01
*BIRC5*	2.07	3.58	2.85	0.00
***EPCAM***	10.14	11.53	2.63	0.00
***FBP1***	7.29	8.65	2.58	0.00
***EGF***	3.70	4.93	2.35	0.00
***ODC1***	10.54	11.68	2.21	0.00
***TOP2A***	5.84	6.98	2.21	0.00
*GLDC*	1.39	2.50	2.16	0.01
***SLC7A1***	7.77	8.86	2.13	0.00
*EZH2*	5.38	6.42	2.05	0.00
*CD24*	8.43	9.44	2.01	0.00

^1^ Ranked on fold change. ^2^ Fold change calculated as 2^(log2 Mean FPM value PCa (LG-PCa and HG-PCa) − log2 Mean FPM value no PCa (Normal and BPH))^. ^3^ Adjusted *p*-value < 0.05 (Mann–Whitney U test). Gene names are presented in bold if they are also identified as significant in microarray analysis.

**Table 2:** Microarray analysis results for the top 25 of the 270 genes with expression levels that are significantly higher in PCa compared to no PCa and with a fold change > 2.

	Mean 2log Value per Group			
Gene ^1,2^	No PCa (Normal and BPH)	PCa (LG-PCa and HG-PCa)	Fold Change ^3^	Adjusted *p*-Value ^4^
*CRISP3*	3.70	7.53	14.25	0.00
*OR51E2*	7.29	10.86	11.81	0.00
*AMACR*	6.73	10.02	9.80	0.00
*TDRD1*	3.34	6.62	9.71	0.00
*ACSM1*	3.99	6.99	7.98	0.00
*THBS4*	4.47	7.28	6.97	0.00
*GDF15*	7.01	9.77	6.77	0.00
*TRGV9*	6.35	9.10	6.74	0.00
*RPS24*	8.81	11.51	6.50	0.00
*RPS29*	8.73	11.37	6.26	0.00
*GLYATL1*	4.83	7.47	6.23	0.00
*TMEM45B*	5.14	7.76	6.15	0.00
*SLC25A33*	4.96	7.55	6.04	0.00
*AGR2*	6.86	9.30	5.43	0.00
***PLA1A***	5.30	7.71	5.31	0.00
*CLDN8*	5.88	8.28	5.29	0.00
*GCNT1*	5.50	7.89	5.25	0.00
***ERG***	5.00	7.39	5.23	0.01
*TMSB15A*	4.15	6.52	5.16	0.00
*C11orf98*	4.72	7.08	5.15	0.00
*ASPN*	4.69	6.99	4.90	0.00
*RRM2*	4.14	6.43	4.87	0.00
*GOLM1*	8.23	10.50	4.82	0.00
***FOLH1***	6.66	8.86	4.60	0.00
*SFRP4*	6.28	8.44	4.45	0.00

^1^ Ranked on fold change. ^2^ For the complete list of 270 genes, see Table S1. ^3^ Fold change calculated as 2^(mean 2log value PCa (LG-PCa and HG-PCa) − mean 2log value no PCa (Normal and BPH))^. ^4^ Adjusted *p*-value < 0.05 (Mann–Whitney U test). Gene names are presented in bold if they are also identified as significant in smMIP analysis.

**Table 3:** smMIP analysis results for the 8 genes with expression levels that are significantly higher in HG-PCa compared to normal, BPH or LG-PCa and with a fold change >2.

	Mean FPM Log2 per Group			
Gene ^1^	Normal, BPH and LG-PCa	HG-PCa	Fold Change ^2^	Adjusted *p*-Value ^3^
*HOXC6*	3.36	5.29	3.81	0.04
***CLDN3***	3.82	5.39	2.97	0.04
*ALOX15*	1.12	2.54	2.69	0.04
*LNCHOXC8*	2.00	3.43	2.69	0.04
***FOLH1***	11.39	12.76	2.57	0.04
*BIRC5*	2.68	3.86	2.26	0.04
***FAP***	5.82	6.92	2.14	0.04
*CLDN4*	6.52	7.55	2.04	0.05

^1^ Ranked on fold change. ^2^ Fold change calculated as 2^(log2 Mean FPM value HG-PCa) − log2 Mean FPM value no PCa or LG-PCa (Normal, BPH and LG-PCa))^. ^3^ Adjusted *p*-value < 0.05 (Mann–Whitney U test). Gene names are presented in bold if they are also identified as significant in microarray analysis.

**Table 4:** Microarray analysis results for the 17 genes with expression levels that are significantly higher in HG-PCa compared to no PCa, BPH or LG-PCa and with a fold change >2.

	Mean 2log Value per Group			
Gene ^1^	Normal, BPH and LG-PCa	HG-PCa	Fold Change ^2^	Adjusted *p*-Value ^3^
*MYL2*	3.83	5.80	3.92	0.04
*AMACR*	8.26	10.15	3.71	0.03
*RRM2*	5.10	6.73	3.09	0.02
*ASPN*	5.67	7.26	2.99	0.02
***FOLH1***	7.63	9.06	2.69	0.05
*MS4A8*	4.59	6.01	2.68	0.03
*CLDN8*	7.01	8.34	2.52	0.04
*COMP*	4.97	6.24	2.41	0.02
*TRIB1*	8.57	9.82	2.37	0.04
*ND6*	8.96	10.17	2.31	0.02
*CHRM3*	4.79	5.99	2.29	0.04
*PPFIA2*	4.16	5.35	2.27	0.04
*RPL35*	3.32	4.46	2.21	0.03
*ITGBL1*	5.28	6.39	2.15	0.03
***CLDN3***	7.90	8.97	2.09	0.02
*SHISA2*	5.06	6.10	2.06	0.03
***FAP***	4.68	5.70	2.02	0.02

^1^ Ranked on fold change. ^2^ Fold change calculated as 2^(mean 2log value HG-PCa − mean 2log value no PCa or LG-PCa (Normal, BPH and LG-PCa))^. ^3^ Adjusted *p*-value < 0.05 (Mann–Whitney U test). Gene names are presented in bold if they are also identified as significant in smMIP analysis.

## Data Availability

Data are not available due to restrictions (legal).

## Supplementary Materials

The supporting information can be downloaded at: https://www.mdpi.com/article/10.3390/ijms252211907/s1.
