# Supplementary material for: Discovery of RNA Biomarkers for Prostate Cancer Using Cross-Platform Transcriptomics
Source: Int J Mol Sci. 2024 Nov 6;25(22):11907. doi: 10.3390/ijms252211907 (PMC11593515; doi:10.3390/ijms252211907)
Supplement: Supplementary file 1 [file ijms-25-11907-s001.zip › ijms-3287135-supplementary.pdf]

Supporting information S1

Table S1 File. Results microarray analysis. This table shows mean 2log values, fold change values and adjusted p-values for the 270 genes with expression levels that were significantly higher in PCa (both LG-PCa and HG-PCa) compared to no PCa (both no PCa and BPH) (adjusted p-value: <.05) and with a fold change >2. Ranked on fold change. Fold change calculated as $2^{\text{(mean 2log value PCa (LG-PCa and HG-PCa) - mean 2log value no PCa (No PCa and BPH)}}$. Gene names are presented in bold when they are also identified as significant in smMIPs analysis.

	Mean 2log value			
**Gene**	**No PCa (no-PCa and BPH)**	**PCa (LG-PCa and HG-PCa)**	**Fold change**	**Adjusted p-value**
CRISP3	3.70	7.53	14.25	0.00
OR51E2	7.29	10.86	11.81	0.00
AMACR	6.73	10.02	9.80	0.00
TDRD1	3.34	6.62	9.71	0.00
ACSM1	3.99	6.99	7.98	0.00
THBS4	4.47	7.28	6.97	0.00
GDF15	7.01	9.77	6.77	0.00
TRGV9	6.35	9.10	6.74	0.00
RPS24	8.81	11.51	6.50	0.00
RPS29	8.73	11.37	6.26	0.00
GLYATL1	4.83	7.47	6.23	0.00
TMEM45B	5.14	7.76	6.15	0.00
SLC25A33	4.96	7.55	6.04	0.00
AGR2	6.86	9.30	5.43	0.00
**PLA1A**	5.30	7.71	5.31	0.00
CLDN8	5.88	8.28	5.29	0.00
GCNT1	5.50	7.89	5.25	0.00
**ERG**	5.00	7.39	5.23	0.01
TMSB15A	4.15	6.52	5.16	0.00
C11orf98	4.72	7.08	5.15	0.00
ASPN	4.69	6.99	4.90	0.00
RRM2	4.14	6.43	4.87	0.00
GOLM1	8.23	10.50	4.82	0.00
**FOLH1**	6.66	8.86	4.60	0.00
SFRP4	6.28	8.44	4.45	0.00
HPN	4.69	6.84	4.44	0.00
ND6	8.00	10.07	4.20	0.00
TRIB1	7.63	9.70	4.19	0.00
THYN1	4.26	6.31	4.13	0.00
NPY	7.41	9.40	3.98	0.00
MS4A8	3.76	5.74	3.94	0.00
HIST3H2A	4.92	6.90	3.93	0.00
TMTC4	6.11	8.08	3.90	0.00

**CLDN4**	7.88	9.82	3.85	0.00
GADD45G	5.90	7.82	3.79	0.00
**FASN**	7.78	9.66	3.68	0.00
SLC7A11	4.28	6.16	3.68	0.00
UAP1	6.47	8.34	3.65	0.00
**MYC**	7.06	8.91	3.61	0.00
POPDC3	3.03	4.85	3.53	0.00
PGC	5.93	7.75	3.53	0.00
STX19	3.26	5.05	3.44	0.00
STEAP4	8.57	10.33	3.39	0.00
NKAIN1	3.88	5.64	3.39	0.00
F5	4.84	6.60	3.38	0.01
CLDN7	6.61	8.36	3.36	0.00
APOF	3.20	4.94	3.35	0.00
TRIM36	5.53	7.28	3.35	0.00
CENPN	5.03	6.77	3.32	0.00
DDT	6.55	8.28	3.32	0.00
STEAP1	6.42	8.14	3.29	0.00
RPS2	5.33	7.05	3.29	0.00
LUZP2	5.26	6.96	3.25	0.00
SLC38A11	5.37	7.05	3.22	0.00
SERPINE1	6.31	8.00	3.22	0.00
VSTM2L	5.20	6.88	3.21	0.00
SIM2	5.08	6.76	3.19	0.00
SLC43A1	6.05	7.71	3.18	0.00
ALDH3B2	4.01	5.67	3.16	0.00
TDO2	3.13	4.37	2.37	0.13
CHRM3	4.10	5.75	3.14	0.00
CGREF1	4.32	5.95	3.11	0.00
OR51T1	3.25	4.89	3.10	0.00
SERHL	4.19	5.82	3.09	0.00
BHLHA15	5.24	6.86	3.08	0.00
GJB1	4.86	6.46	3.04	0.00
C19orf48	6.76	8.37	3.03	0.00
GPR160	7.41	9.01	3.01	0.00
PLA2G2A	8.79	10.38	3.01	0.00
PRAC1	6.27	7.86	3.00	0.00
**FBP1**	6.87	8.44	2.97	0.00
TUBB2A	4.98	6.54	2.96	0.00
LYPLA1	6.23	7.80	2.95	0.00
TLCD1	4.35	5.89	2.92	0.00
DNAH5	4.48	6.02	2.91	0.00
NETO2	4.32	5.86	2.91	0.00
CXCL11	4.52	6.06	2.90	0.00
SDK1	5.00	6.52	2.86	0.00
KCNH8	3.80	5.32	2.86	0.00
ARHGEF38	6.77	8.28	2.85	0.00
MAGEB17	3.46	4.98	2.85	0.00
OR51F2	3.55	5.05	2.83	0.00
RPL11	9.47	10.97	2.83	0.00
PTP4A3	6.92	8.42	2.82	0.00
DNASE2B	3.99	5.48	2.80	0.00
VPS37C	4.42	5.91	2.79	0.00
**CLDN3**	7.28	8.76	2.79	0.00
PDIA5	6.87	8.35	2.79	0.00
RPS15A	6.41	7.89	2.79	0.00
**DLX1**	4.39	5.86	2.78	0.00
FFAR2	2.85	4.32	2.76	0.00
SEC61G	7.86	9.32	2.75	0.00
PTRHD1	6.27	7.73	2.75	0.00

SLC27A2	5.64	7.09	2.74	0.00
CBR3	4.37	5.82	2.73	0.00
NEK5	3.51	4.93	2.69	0.00
SMPDL3B	5.12	6.54	2.69	0.00
**EPCAM**	7.40	8.82	2.67	0.00
C4B	7.51	8.92	2.66	0.00
TUBA1C	6.24	7.65	2.65	0.00
ITGBL1	4.73	6.13	2.65	0.00
HIST1H3C	4.76	6.16	2.64	0.00
CXCL14	4.96	6.35	2.63	0.00
MYL2	3.56	4.96	2.63	0.00
RAP1GAP	5.79	7.18	2.61	0.00
TMEM178A	5.85	7.23	2.61	0.00
GALNT7	8.20	9.58	2.60	0.00
CACNA1D	6.58	7.96	2.60	0.00
KCNG3	3.73	5.10	2.59	0.00
TSTA3	7.76	9.13	2.59	0.00
RAB3B	7.68	9.04	2.57	0.00
CREB3L1	6.65	8.01	2.57	0.00
ANKRD34B	2.96	4.32	2.57	0.00
OCIAD2	6.19	7.54	2.56	0.00
UBC	9.97	11.33	2.56	0.00
PART1	6.09	7.43	2.53	0.00
SYCE1L	5.40	6.74	2.53	0.00
RHPN2	6.88	8.20	2.50	0.00
MYO6	8.05	9.38	2.50	0.00
BAMBI	6.17	7.49	2.50	0.00
**HOXC6**	3.64	4.95	2.49	0.00
TMC5	7.07	8.39	2.49	0.00
AIMP2	5.94	7.26	2.48	0.00
GAL	3.40	4.71	2.47	0.00
PTPRT	3.84	5.15	2.47	0.00
ABRACL	5.61	6.91	2.47	0.00
ACY1	5.81	7.11	2.45	0.00
SPR	5.95	7.25	2.45	0.00
SFXN2	5.88	7.18	2.45	0.00
RAB17	5.00	6.29	2.45	0.00
TSPAN13	8.66	9.93	2.42	0.00
SLC5A1	4.73	6.00	2.42	0.00
PLA2G7	8.68	9.95	2.41	0.00
PCSK6	4.45	5.72	2.41	0.00
GGCT	6.74	8.01	2.41	0.00
PCDHB2	5.54	6.81	2.41	0.01
RPL35	2.87	4.13	2.40	0.00
C5orf30	4.66	5.92	2.39	0.00
C15orf48	5.01	6.26	2.38	0.00
C8orf59	7.73	8.98	2.38	0.00
NCL	7.48	8.72	2.36	0.00
ABHD11	5.21	6.44	2.35	0.00
SLC6A14	4.90	6.13	2.34	0.01
DDX21	6.95	8.18	2.34	0.00
ABCC4	9.15	10.36	2.32	0.00
PPFIA2	3.76	4.97	2.31	0.02
B3GAT1	4.08	5.29	2.31	0.00
**ALOX15**	3.74	4.93	2.29	0.00
MB	4.84	6.03	2.28	0.00
AIFM1	5.49	6.67	2.28	0.00
SERINC4	3.70	4.88	2.28	0.00
BIK	4.59	5.78	2.27	0.00
LRRN1	6.34	7.52	2.27	0.01

NDUFB10	6.41	7.59	2.27	0.00
SLIT1	3.98	5.16	2.27	0.00
SLC25A25	5.46	6.64	2.27	0.00
SPTBN2	5.50	6.68	2.27	0.00
TRAF4	6.79	7.97	2.27	0.00
ELAVL2	3.14	4.32	2.26	0.00
ELF5	4.28	5.45	2.26	0.00
PRSS8	7.03	8.20	2.26	0.00
SLCO1A2	3.23	4.40	2.25	0.00
IMPDH2	7.96	9.13	2.25	0.00
CBLC	5.03	6.19	2.24	0.00
SLC45A2	3.37	4.53	2.24	0.00
ATP8A2	3.25	4.41	2.24	0.00
TFF3	5.77	6.92	2.23	0.00
PRDX4	6.46	7.61	2.22	0.00
ST14	7.25	8.40	2.22	0.00
RASD1	6.69	7.83	2.21	0.00
LIPH	6.28	7.42	2.21	0.00
ZNF552	5.31	6.46	2.21	0.00
GRIN3A	3.04	4.18	2.21	0.00
CAMKK2	6.52	7.66	2.21	0.00
**SOX14**	3.95	5.09	2.20	0.00
NME1-NME2	7.27	8.41	2.20	0.00
SLCO1B3	3.24	4.38	2.20	0.00
DOPEY2	6.25	7.39	2.20	0.00
SCGN	3.94	5.08	2.20	0.00
ZNF511	5.96	7.10	2.19	0.00
NUDT8	5.36	6.49	2.19	0.00
SLC44A5	3.73	4.86	2.19	0.02
SPON2	7.65	8.78	2.18	0.02
GABRB3	5.82	6.94	2.17	0.00
ARFGEF3	8.15	9.27	2.17	0.00
CTNND2	4.52	5.64	2.17	0.00
RAP1B	6.16	7.28	2.17	0.00
SLC25A10	5.44	6.55	2.17	0.00
PRR16	5.33	6.44	2.17	0.00
CSRNP1	5.94	7.05	2.17	0.00
PSMB5	6.21	7.33	2.17	0.00
AP1M2	7.24	8.35	2.16	0.00
LRRC59	7.88	8.99	2.16	0.00
E2F5	4.70	5.81	2.16	0.00
**ODC1**	10.07	11.17	2.16	0.00
MYRIP	4.05	5.16	2.15	0.00
FAM3D	5.25	6.35	2.15	0.00
**SLC7A1**	6.97	8.07	2.15	0.00
TMEM141	6.96	8.06	2.15	0.00
ADRB1.1	6.60	7.70	2.15	0.00
NCALD	5.69	6.79	2.14	0.00
CHDH	5.66	6.75	2.14	0.00
UGT2B4	3.07	4.17	2.13	0.00
TUFT1	5.37	6.46	2.13	0.00
TRPM4	7.10	8.19	2.13	0.00
BEND4	5.67	6.75	2.12	0.00
CYP2J2	4.64	5.72	2.12	0.00
RGS17	3.19	4.26	2.10	0.00
F3	7.22	8.29	2.10	0.00
SAT1	9.80	10.87	2.10	0.00
SH3RF1	7.61	8.68	2.10	0.00
CYP1B1	7.62	8.69	2.10	0.01
COMP	4.69	5.76	2.10	0.00

POLR2H	8.18	9.24	2.10	0.00
RND1	4.81	5.88	2.09	0.00
APMAP	7.15	8.22	2.09	0.00
SMCO4	6.72	7.78	2.09	0.00
AK5	3.83	4.90	2.09	0.00
HOOK2	4.92	5.98	2.09	0.00
WWC1	5.64	6.71	2.09	0.00
EFNA1	6.25	7.31	2.09	0.00
TSPAN1	10.44	11.50	2.08	0.00
MRPL41	8.19	9.24	2.08	0.00
DUS1L	6.57	7.63	2.08	0.00
MAFK.1	5.10	6.15	2.08	0.00
**TOP2A**	4.00	5.06	2.08	0.00
NUPR1	8.76	9.82	2.08	0.00
NDUFA1	7.51	8.57	2.08	0.00
SMPD2	6.33	7.38	2.07	0.00
EGR2	6.35	7.41	2.07	0.00
CYTH2	6.70	7.75	2.07	0.00
TKT	7.09	8.14	2.06	0.00
PEX10	5.97	7.02	2.06	0.00
**EGF**	4.53	5.57	2.06	0.00
LSM7	5.09	6.13	2.06	0.00
PKP3	4.98	6.02	2.06	0.00
FAM50A	5.77	6.81	2.06	0.00
SPDEF	8.41	9.44	2.05	0.00
MYBPC1	7.78	8.82	2.05	0.00
RUVBL1	5.32	6.36	2.05	0.00
NME1	6.66	7.70	2.05	0.00
NUDT9	5.42	6.46	2.05	0.00
MLPH	7.63	8.66	2.04	0.00
FAAH	7.43	8.46	2.04	0.00
YPEL1	4.63	5.66	2.04	0.00
C14orf166	7.30	8.33	2.04	0.00
RPF2	7.57	8.60	2.04	0.00
PIGW	4.70	5.72	2.03	0.00
KLF6	7.25	8.27	2.03	0.00
DND1	6.24	7.26	2.03	0.00
GMDS	7.14	8.16	2.03	0.00
AGR3	3.22	4.24	2.03	0.00
UQCRQ	8.10	9.11	2.02	0.00
ERRFI1	7.23	8.24	2.02	0.00
PRC1	4.07	5.08	2.02	0.00
HID1	7.04	8.05	2.02	0.00
POP7	6.12	7.13	2.02	0.00
TPX2	3.64	4.66	2.02	0.00
ATF3	6.41	7.42	2.01	0.02
DSC2	7.40	8.40	2.01	0.00
HIST1H3B	5.19	6.20	2.01	0.00
PODXL2	5.38	6.39	2.01	0.00
RPS14	4.32	5.33	2.01	0.00
ZNF233	4.43	5.43	2.01	0.00
PCDHB8	3.87	4.88	2.01	0.01
RUVBL2	6.69	7.69	2.01	0.00
DUSP5	6.09	7.09	2.01	0.00
COX7A2	8.53	9.53	2.00	0.00
RPSAP58	4.42	5.42	2.00	0.00
CORO1B	7.02	8.02	2.00	0.00
GUSB	7.41	8.41	2.00	0.00

Supporting information S2

S2 File. Biomarkers identified with microarray only. Overview of the 250 genes that were identified by the microarray analysis as potential marker that can discriminates between PCa (both LG-PCa and HG-PCa) and no PCa (both no PCa and BPH) which were not identified by the smMIPs analysis (these genes were not evaluated by the smMIPs method).

SLC25A33, RPL11, SMPDL3B, TSPAN1, FAAH, AK5, DNASE2B, TUFT1, EFNA1, UAP1, ATF3, CHRM3, PEX10, ERRFI1, PLA2G2A, RAP1GAP, NKAIN1, RAB3B, CYP2J2, SLC44A5, F3, F5, HIST3H2A, RRM2, TMEM178A, SPR, MLPH, PTRHD1, CGREF1, CYP1B1, KCNG3, SLC38A11, NCL, RAB17, LRRN1, KCNH8, MYRIP, ACY1, CACNA1D, PDIA5, PODXL2, GPR160, POLR2H, CSRNP1, IMPDH2, TKT, CHDH, FAM3D, STX19, RUVBL1, LIPH, NUDT9, ARHGEF38, GALNT7, SPON2, BEND4, OCIAD2, UGT2B4, CXCL11, SLC7A11, SH3RF1, PART1, THBS4, C5orf30, PRR16, UQCRQ, PCDHB2, PCDHB8, WWC1, CTNND2, DNAH5, SLC45A2, AMACR, ANKRD34B, TRIM36, CXCL14, DND1, RPS14, SCGN, HIST1H3C, C4B, MYO6, SMPD2, RPF2, ARFGEF3, ABRACL, GMDS, TUBB2A, HIST1H3B, SPDEF, PGC, PLA2G7, CRISP3, COX7A2, POPDC3, RGS17, MAFK.1, SDK1, AIMP2, TSPAN13, NPY, STEAP1, BHLHA15, POP7, SERPINE1, AGR2, AGR3, GGCT, SFRP4, TRGV9, SEC61G, GUSB, ABHD11, STEAP4, E2F5, TRIB1, PTP4A3, C8orf59, NCALD, TSTA3, GCNT1, GADD45G, SLC25A25, TMEM141, MRPL41, ELAVL2, GOLM1, ASPN, GRIN3A, RPL35, BAMBI, DDX21, RPS24, SFXN2, DUSP5, ADRB1.1, TDRD1, ZNF511, KLF6, EGR2, SLIT1, PKP3, OR51F2, OR51T1, LUZP2, CREB3L1, GLYATL1, MS4A8, GAL, TMEM45B, ST14, OR51E2, ELF5, SLC43A1, VPS37C, C11orf98, SPTBN2, CORO1B, NUDT8, ALDH3B2, SMCO4, THYN1, B3GAT1, SLCO1B3, TUBA1C, RAP1B, MYBPC1, SLCO1A2, RND1, APOF, PPFIA2, MYL2, CAMKK2, UBC, ATP8A2, ITGBL1, NEK5, ABCC4, TMTC4, C14orf166, PSMB5, RPS29, C15orf48, SLC27A2, GABRB3, SERINC4, PRC1, PCSK6, NDUFB10, TMC5, NUPR1, SYCE1L, CENPN, RPS2, RPS15A, ACSM1, PRSS8, NETO2, TRAF4, PIGW, NME1, NME1-NME2, SLC25A10, CLDN7, RASD1, TLCD1, PRAC1, LRRC59, HID1, DUS1L, DSC2, GDF15, RPSAP58, HPN, FFAR2, ZNF233, CBLC, CYTH2, TRPM4, LSM7, AP1M2, HOOK2, COMP, RHPN2, C19orf48, ZNF552, TPX2, VSTM2L, APMAP, PTPRT, CBR3, DOPEY2, SIM2, CLDN8, TFF3, SERHL, BIK, YPEL1, DDT, MB, MAGEB17, PRDX4, SAT1, GJB1, SLC6A14, NDUFA1, FAM50A, TMSB15A, AIFM1, ND6.
